# Genomic Signatures Underlying Environmental Adaptation and Reproductive Traits in the Tibetan Pig

**DOI:** 10.3390/ani16030509

**Published:** 2026-02-05

**Authors:** Mengqi Duan, Songyuan Zhang, Hang Jiao, Peng Shang, Chunli Li, Kejun Wang

**Affiliations:** 1 College of Animal Science and Technology, Henan Agricultural University, Zhengzhou 450046, China; zduanduan0117@163.com (M.D.); songyuanzhang2022@163.com (S.Z.); jiaohang0811@163.com (H.J.); 2 College of Animal Science, Xizang Agriculture and Animal Husbandry University, Linzhi 860000, China; nemoshpmh@126.com

**Keywords:** whole-genome resequencing, genome assembly, structural variation, Tibetan pig

## Abstract

Background: The Tibetan pig, a highland breed with exceptional adaptability to harsh environments (cold, hypoxia, coarse feed) but poor growth/reproductive traits, was studied to uncover genetic mechanisms and support breeding improvements. Methods: We conducted de novo genome assembly of a male Tibetan pig using stLFR sequencing, supplemented with ONT data, and compared the assembly to the Duroc pig genome (v11.1). Results: The assembled genome (2.25 Gb, contig N50 = 136.5 Mb, GC content = 41.74%, 94.16× coverage) showed 96.9% BUSCO completeness. Structural variant (SV) analysis identified 22,008 insertions and 27,639 deletions, with an SV genotyping accuracy of 0.9735. Selective sweep analysis highlighted adaptive genes: *XIRP2* (cardiac function), *KSR2*/*CACNA1A* (fat metabolism), *COL11A1* (cartilage), and *ADORA2A* (vascular regulation). Tibetan pigs exhibited the fewest and shortest runs of homozygosity (ROHs) among four breeds, with ROH-linked SNPs implicating lipid catabolism genes (*LIPE*, *PNPLA2*, *MGLL*, *DGAT1*). An SNP-based GWAS revealed reproductive trait associations: immune gene *IL2RB*, energy metabolism genes *PRKAG2*, *ADGRA1*, and *PTPRN2*, and growth genes *SLIT2* and *BMP6*. SV analysis identified additional candidates: energy metabolism genes *HAO2* and *NRG4*, growth genes *MTUS2* and *FGF12*, and immune genes *SCGB1A1* and *C8A*. Conclusions: This study provides a chromosome-level genome assembly of a male Tibetan pig (generated from stLFR and ONT data), and, through whole-genome resequencing of 124 Tibetan sows, identifies key genetic factors underlying Tibetan pigs’ environmental adaptability and reproductive limitations, enabling genomic strategies to enhance breeding efficiency while preserving adaptive traits.

## Simple Summary

The Tibetan pig is a unique breed adapted to the harsh high-altitude environment of the Qinghai–Tibet Plateau, exhibiting strong tolerance to cold, hypoxia, and coarse feed, but with relatively low reproductive performance. To uncover the genetic basis of these traits, we constructed a high-quality chromosome-level genome of a male Tibetan pig and analyzed whole-genome resequencing data from 124 sows. By comparing Tibetan pigs with lowland breeds, we identified key genetic regions and genes linked to environmental adaptation—such as those involved in heart function, fat metabolism, and blood vessel regulation. We also discovered genes associated with reproductive traits like litter size and piglet weight, which are related to immune response, energy metabolism, and growth. These findings provide new insights into the genetic mechanisms of high-altitude adaptation and offer valuable markers for breeding programs aimed at improving Tibetan pig productivity while preserving their hardy characteristics.

## 1. Introduction

Tibetan pigs are primarily distributed in the Qinghai–Tibet Plateau region, represent a typical plateau miniature pig breed, and hold special significance in pig genetic resource conservation and utilization [1]. Moreover, as one of the major economic animals in Tibet, Tibetan pigs play a crucial role in the development of the local agricultural and pastoral economy [2]. Tibetan pigs, a breed indigenous to China’s high-altitude regions, have evolved under persistent selective pressures from hypoxia and extreme cold. Consequently, they have developed a suite of distinctive and heritable traits, including adaptations to high-altitude hypoxia [3], robust disease resistance [4], enhanced fat deposition capacity [5], and relatively low reproductive performance with a litter size of only 5–8 piglets per farrowing [6]. This unique ecological niche has likely shaped their genetic architecture, resulting in characteristically low reproductive performance—a common adaptive trade-off observed in high-altitude species. Furthermore, the very environmental factors that drove their specialization—most notably hypoxia adaptation and cold resistance—now present significant obstacles for conventional breeding and crossbreeding programs. This is because the physiological and metabolic adaptations that ensure survival in harsh plateau environments can directly impair reproductive efficiency and breeding outcomes. For instance, chronic hypoxia is known to cause damage to the reproductive system, adversely affecting hormone levels, gonadal development, and gamete motility [7,8]. Similarly, cold stress can disrupt the estrous cycle, cause hormonal disorders, and impair ovarian and uterine microcirculation [9]. These inherent physiological constraints limit the reproductive performance of Tibetan pigs under intensive breeding conditions and complicate crossbreeding efforts, as introducing genes for higher productivity from lowland breeds may further destabilize these finely tuned adaptive mechanisms, leading to reduced hybrid viability or loss of the core adaptive traits.

With the rapid advancement of genomics technologies, genome-wide association studies (GWASs) and genomic selection (GS) have been widely applied to explore the germplasm characteristics of Tibetan pigs [10]. However, current research primarily focuses on genetic effect analysis at the level of single-nucleotide polymorphisms (SNPs) and small insertions/deletions (indels). Researchers aim to improve trait prediction accuracy by optimizing reference population construction strategies and refining statistical models [11]. Yet, these models often implicitly assume that “all SNPs contribute equally to genetic variation,” making it difficult to fully unravel the heterogeneity and complexity of the genetic architecture underlying hypoxia adaptation [12]. In recent years, SNP/indel-based studies have indeed provided important clues for understanding the genetic characteristics of Tibetan pigs. For instance, population genetics analyses have revealed the central position of the Tibetan pig population within the overall genetic diversity of Chinese pigs. For example, whole-genome resequencing was performed on 10 geographically isolated Tibetan pig populations. Analyses of genetic diversity and allelic diversity revealed that the Tibetan pig population in Tibet contributed the most to the meta-population. Based on this, it is inferred that the Tibetan pig population in Tibet has the lowest degree of genetic differentiation and serves as a gene flow hub for the genetic diversity within these 10 populations [13]. Through selective sweep scans, candidate genes (such as *HIF3A*, *RCN3*, *HIGD2A*, *PCK2*, *IRF9*) and their associated SNVs/INDELs related to cardiopulmonary function, fat metabolism, and hypoxia adaptation have been identified [10,14]. Comparative genomics and transcriptomics have also uncovered differences in the expression of hypoxia-responsive genes between Tibetan pigs and other pig breeds [15]. Hypoxia adaptation and reproductive performance in Tibetan pigs represent complex polygenic phenotypes. While studies focusing on single-nucleotide polymorphisms (SNPs) and small insertions/deletions (indels) have provided initial insights, such approaches offer a fundamentally limited perspective for both deciphering genetic architectures and enabling effective breeding. This limitation arises because complex adaptive traits are often influenced by a broader spectrum of genomic variations—including structural variants (SVs) and copy number variants (CNVs)—that are notoriously challenging to detect with standard SNP arrays or short-read sequencing. Crucially, an incomplete variant catalog directly hinders genomic selection (GS) programs. Compelling empirical evidence from aquaculture species demonstrates that prediction accuracies for complex growth traits can be significantly higher when models incorporate SV markers compared to those relying solely on SNPs, underscoring how reliance on a limited variant set can limit genetic gain [8]. Therefore, moving beyond an SNP/indel-centric view is imperative. The future of precision breeding lies in integrating multi-omics data to construct a complete functional blueprint, as exemplified by recent research on swine that combines GWASs with transcriptomics to map variant–gene–trait regulatory circuits [7]. Our study on Tibetan pigs is positioned within this integrative framework, aiming to provide a more comprehensive genetic basis for their unique germplasm to overcome existing breeding bottlenecks. In this context, structural variation (SV), as a core type of genomic variation, is increasingly recognized for its importance. Numerous studies have demonstrated that SVs play a decisive role in species’ environmental adaptation, the formation of key economic traits, and disease susceptibility through mechanisms such as altering gene dosage, disrupting/creating regulatory elements, and influencing three-dimensional chromatin structure [16]. Within the Sus genus, SVs have also been confirmed to significantly impact various important traits, including growth, metabolism, immunity, and reproduction. Particularly noteworthy is that integrating SV information holds promise for overcoming the prediction accuracy limitations of current SNP-based models, especially in scenarios involving genetically diverse backgrounds or limited reference population sizes [17]. However, systematic genetic dissection of the high-altitude hypoxia adaptation in Tibetan pigs, particularly in-depth research at the SV level, is scarcely reported. Although pan-genome studies have convincingly demonstrated the extensive and profound contribution of SVs to adaptive evolution [18], the comprehensive SV landscape unique to Tibetan pigs and its association mechanisms with core germplasm characteristics like extreme hypoxia tolerance remain largely unexplored.

To gain a clearer understanding of Tibetan pig germplasm characteristics, this study focuses on Tibetan pigs as the primary research subject and selects three Chinese indigenous pig breeds—Meishan, Erhualian, and Wuzhishan—as comparative groups for whole-genome resequencing data analysis. The rationale for choosing these breeds lies in their representation of environmental adaptability and phenotypic traits that are markedly distinct from those of Tibetan pigs, thereby facilitating the identification of genetic signals unique to Tibetan pigs. Meishan and Erhualian pigs are primarily distributed in the warm, humid, low-altitude Taihu Lake region of eastern China, an area characterized by abundant feed resources [19]. In contrast, the Wuzhishan pig originates from the tropical island mountainous areas of Hainan Province, China, where the climate is perennially hot and rainy, and its small size has garnered significant attention in medical and veterinary research [20]. In terms of reproductive performance, these breeds stand in sharp contrast to Tibetan pigs: for Tibetan primiparous sows, the average Total Number Born (TNB), Number Born Alive (NBA), and litter birth weight are (5.92 ± 1.71) piglets, (5.54 ± 1.76) piglets, and (4.61 ± 1.67) kg, respectively [21]. The Meishan pig is globally recognized as one of the breeds with the highest litter size. Its primiparous sows have an average litter size of 12.7 piglets. In the control group, Large White primiparous sows have an average total litter size of 15.0 piglets. On average, Meishan sows produce 2–3 more live piglets per litter than Large White sows [22]. The Erhualian pig averages over 15 piglets per litter, with some high-yielding individuals exceeding 20 piglets, far surpassing Western commercial breeds [23,24]. In comparison, Wuzhishan sows have an average TNB of 7.68 piglets and an NBA of 6.72 piglets, exhibiting characteristics such as slow growth and low reproductive rates, but strong disease resistance [25]. Additionally, Wuzhishan pigs are small in size, reach sexual maturity early, and exhibit stable genetics, and microsatellite analyses indicate high genetic diversity [26]. These traits are closely linked to their respective ecological environments, reflecting the rich genetic diversity and adaptive features of Chinese indigenous pig breeds. By comparing Tibetan pigs with these breeds, which differ significantly in reproductive capacity, growth patterns, and environmental adaptability, we can more effectively identify genetic regions and variations specifically associated with Tibetan pigs’ plateau adaptation and low reproductive performance.

This study employs population genetics methods to analyze key genes involved in the development of Tibetan pigs’ environmental adaptation capabilities. We constructed a Tibetan pig genome and identified chromosomal structural variations between Tibetan pigs and the reference genome. Structural variations and single-nucleotide polymorphisms were utilized to identify important variants associated with Tibetan pig reproductive traits. This research aims to provide a theoretical foundation for elucidating the adaptive mechanisms underlying Tibetan pig germplasm characteristics and supporting the development of the related industry.

## 2. Materials and Methods

### 2.1. Animals and Sample Preparation

This study utilized a total of 165 pig samples, comprising 124 Tibetan pigs, 11 Meishan pigs, 13 Wuzhishan pigs, and 17 Erhualian pigs. Among these, the 124 Tibetan pigs were 1–2-year-old sows from a pig farm in Nyingchi, Tibet, China. Ear tissue samples were collected, preserved in 75% ethanol (Sinopharm Chemical Reagent Co., Ltd., Shanghai, China), and stored at −20 °C until DNA extraction. Whole-genome resequencing data for the Meishan, Wuzhishan, and Erhualian pigs were obtained from the NCBI database (National Center for Biotechnology Information, Bethesda, MD, USA), with detailed population information provided in Supplementary Table S1.

### 2.2. Determination of Phenotypic Characteristics

This study collected first-parity reproductive records from 124 Tibetan sows, including Total Number Born (TNB), Number Born Alive (NBA), Number of Piglets Weaned (NPW), Weight of Total Number Born (WTNB), and Weaning Litter Weight (WLW). The collected phenotypic data were initially organized using Excel 2021. A total of 443 filtered records were used to calculate the mean, standard deviation, median, minimum, maximum, skewness, and kurtosis. Subsequently, the normality of the phenotypic distribution was assessed using the R package rMVP (v1.4.6).

### 2.3. DNA Extraction, Library Preparation, and Sequencing

De novo genome assembly was based entirely on a specific dataset from a male Tibetan pig. The dataset consists of two components: (a) newly generated stLFR sequencing data for initial assembly and (b) published ONT long-read data obtained from the public database (CNCB-NGDC) for scaffold improvement and gap filling. The DNA extraction, library construction, and stLFR (single-tube long fragment read) sequencing for Tibetan pigs were performed by BGI Genomics (Shenzhen, China). The stLFR co-barcoded DNA libraries were constructed using the MGIEasy stLFR Library Prep Kit (MGI, Shenzhen, China). Sequencing was conducted on the BGISEQ-500 platform (BGI, Shenzhen, China). All 124 Tibetan pigs were sequenced to a minimum depth of 10× coverage, among which 18 individuals were selected for deeper sequencing at no less than 20× coverage. Oxford Nanopore Technology (ONT) sequencing data for Tibetan pigs were obtained from the CNCB-NGDC database (https://ngdc.cncb.ac.cn/; Project Accession: PRJCA005901). The pig reference genome (Sus scrofa assembly Sscrofa11.1) was downloaded from the ENSEMBL website (https://ftp.ensembl.org/pub/release-109/fasta/sus_scrofa/dna/Sus_scrofa.Sscrofa11.1.dna.toplevel.fa.gz, accessed on 4 October 2024).

### 2.4. Genome Assembly of Tibetan Pigs

This study performed de novo assembly of the Tibetan pig genome using stLFR sequencing data through stLFR denovo software (v1.3), a tool specifically developed for stLFR technology based on the Supernova framework that leverages the De Bruijn graph algorithm and barcode information to enhance the resolution of long repetitive sequences and generate genome assemblies. To address gaps within the assembled scaffolds, TGS-GapCloser (v1.2.1) (https://github.com/BGI-Qingdao/TGS-GapCloser, accessed on 4 October 2024) and the patch command in Ragtag were employed, utilizing Tibetan pig ONT data and the reference genome (Sus scrofa Sscrofa11.1), respectively, with unresolved gaps filled using “N” bases. Genome assembly quality was subsequently evaluated by calculating basic metrics, including genome size, N50, and GC content; assessing read mapping rates via alignment of reads to the assembled genome using minimap2 (v2.1) followed by SAMtools flagstat analysis; and determining genomic completeness using BUSCO software (v5.4.7) against the eukaryotic gene database.

### 2.5. SV Annotation and Population Genotyping of Tibetan Pigs

This study employed SVIM-asm and SYRI [27] to identify four types of structural variations (SVs)—insertions, deletions, inversions, and duplications—between the assembled Tibetan pig genome and the Sus scrofa reference genome (Sscrofa11.1). To ensure SV accuracy, SURVIVOR [28] was used to merge SVs jointly detected by both tools, applying the following criteria: SVs > 50 bp in length, identical SV type and orientation, and a maximum distance of 1000 bp between merged SVs. The identified SVs were annotated using ANNOVAR (v2019Oct24) to determine their functional genomic positions. Subsequently, SVtyper was utilized to perform population SV genotyping across the Tibetan pigs, using the SURVIVOR-merged SVs as a template. For sequencing individuals with 20× depth, the seqtk tool (v1.5) (https://github.com/lh3/seqtk, accessed on 4 October 2024) was employed to extract reads corresponding to varying depths and generate subsampled BAM files. Following SV genotyping on these BAM files, truvari (v5.4.0) (https://github.com/ACEnglish/truvari, accessed on 4 October 2024) was used to assess SV genotyping accuracy at different sequencing depths.

### 2.6. Genotyping and Quality Control

This study performed sequence alignment of the resequencing data using the Sus scrofa reference genome from ENSEMBL as the reference. Alignment was conducted with BWA (v0.7.17) using default parameters. After aligning the resequencing data to the reference genome, SAM files were generated and subsequently converted to BAM format using SAMtools (v1.17). SNP variant detection was performed on the resequencing data using GATK (v4.4.0.0). The HaplotypeCaller module in GATK was applied to the aligned BAM files to generate individual gVCF files. All individual gVCF files were then merged using the CombineGVCFs command. To obtain more accurate genotypes for each individual, the GenotypeGVCFs command was used for population variant calling, followed by variant filtration using the VariantFiltration command to filter the detected SNPs. Through these steps, a final VCF file containing SNP variant information for Tibetan pigs was obtained. SNP data underwent quality control using PLINK (v1.9) with the following criteria: SNP call rate > 90%; minor allele frequency (MAF) > 0.01; removal of duplicate SNP loci; and retention of only autosomes (chromosomes 1–18) for subsequent analysis.

### 2.7. Analysis of the Population Genetic Structure of Tibetan Pigs

This study conducted principal component analysis (PCA) using the pca command in PLINK software to evaluate the characteristic vectors of Tibetan pigs, represented by PC1, PC2, PC3, etc. The top two eigenvectors (PC1 and PC2) were visualized using the ggplot2 package (v4.0.2) (https://github.com/tidyverse/ggplot2, accessed on 10 October 2024) in R. The VCF file was converted to PHYLIP format using vcf2phylip.py (v2.8) (https://github.com/edgardomortiz/vcf2phylip, accessed on 10 October 2024). Phylogenetic tree construction was performed with IQ-TREE (v3.0.1) (http://www.iqtree.org/), followed by visual refinement of the tree file using iTOL (v7) (https://itol.embl.de/).

### 2.8. Select Signal Analysis

This study employed common selection signal analysis methods (Fst and XPEHH) to detect signatures of selection in Tibetan pigs compared to Meishan, Wuzhishan, and Erhualian pigs. Vcftools (v0.1.16) was used to calculate FST values across sliding windows, while selscan was applied to compute XPEHH values. Genomic regions ranking in the top 1% of FST windows and the top 1% of XPEHH positive selection signals were annotated using the BioMart tool (http://asia.ensembl.org/biomart/martview/, accessed on 22 October 2024) on the ENSEMBL website to extract gene information within these selected regions. Genes located within the selection regions were defined as candidate selected genes. Genes jointly identified by both methods within the same population were considered the final set of positively selected genes. Functional validation of these genes was conducted through comprehensive literature review.

### 2.9. Analysis of Runs of Homozygosity (ROHs) 

ROH detection was performed using PLINK software via a sliding window approach, calculating the total number, total length, average number, and average length of ROHs per individual. The distribution and frequency of ROHs across autosomes were statistically analyzed at the population level. High-frequency SNPs—defined as those recurrently appearing in ROH segments—were annotated using BioMart in ENSEMBL to extract gene information overlapping these SNPs. Genes containing high-frequency SNPs were designated as target genes. These target genes were subsequently submitted to the KOBAS online database (http://bioinfo.org/kobas, accessed on 22 October 2024) for Kyoto Encyclopedia of Genes and Genomes (KEGG) enrichment analysis, with pathways exhibiting a *p* < 0.05 defined as significantly enriched. To further elucidate gene functions, literature-based functional validation was conducted for genes identified from ROH analyses.

### 2.10. Genome-Wide Association Analysis of Reproductive Traits in Tibetan Pigs

Genome-wide association analysis for SNPs and SVs was conducted using GEMMA software (v0.98.5), employing a univariate linear mixed model (LMM) [29,30]. Prior to the GWAS, the genomic relationship matrix (GRM) between individuals was estimated. The LMM was specified as follows:

*y* = *Wα* + *Xβ* + *u* +*ε*

where *y* is the vector of phenotypic values and *W* refers to the vector of phenotypic value, including covariates such as the top principal components (to control for population stratification), sex, and other relevant factors. *α* is the vector of corresponding coefficients for the fixed effects, including the intercept. *X* is the vector of marker genotypes (for SNPs or SVs) being tested. *β* is the fixed-effect size of the marker. *u* is an *n* × 1 vector of random effects, *u*~MVN*_n_*(0, *λτ*^−1^ *K*) and *ε* is the vector of random residuals, with *ε*~MVN*_n_*(0, *τ*^−1^*ln*). *λ* refers to the ratio between the two variance components; *τ*^−1^ is the variance of the residual errors; *K* is a known *n* × *n* relatedness matrix calculated in a previous step; and I is a known *n* × *n* relatedness matrix calculated in a previous step. MVN*_n_* denotes the *n*-dimensional multivariate normal distribution. Significance of associations between SNPs/SVs and phenotypic traits was assessed using the Wald test.

### 2.11. Annotation of Candidate Genomic Regions

Genes located within 10 kb upstream and downstream of associated SNPs were defined as candidate gene regions. These target regions were annotated using BioMart (v110) in ENSEMBL to extract information on genes within the selection regions, with genes overlapping these regions designated as candidate selected genes. The identified genes were submitted to the KOBAS online database (http://bioinfo.org/kobas, accessed on 25 October 2024) for Kyoto Encyclopedia of Genes and Genomes (KEGG) enrichment analysis. Pathways with a *p* < 0.05 were defined as significantly enriched. To further elucidate gene functions, literature-based functional validation was performed for all annotated genes.

## 3. Results

### 3.1. Tibetan Pig De Novo Assembly

**Figure 1:**
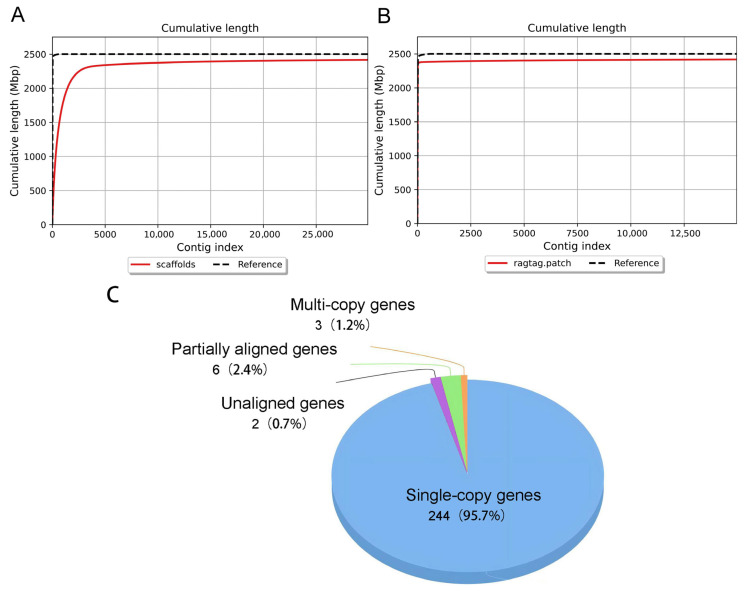
Genome assembly of Tibetan pigs. (**A**) Cumulative contig length curves of the Tibetan pig genome before assembly improvement; (**B**) Cumulative contig length curves of the Tibetan pig genome after assembly improvement; (**C**) Genome Integrity Assessment.

The genome assembly generated from stLFR sequencing data of one male Tibetan pig was 2.28 Gb in size, with an N50 of 1.55 MB, a GC content of 41.74%, and a largest contig length of 1.563 MB. After gap filling, the final assembled genome size was 2.25 Gb, with an N50 of 136.5 MB, a GC content of 41.74%, and a maximum read length of 278.3 MB. The calculated genome coverage of sequencing reads was 94.16%, and the genome completeness assessment revealed a 95.7% proportion of single-copy genes. The statistics for genome assembly metrics before and after scaffolding, along with genome completeness evaluations, are presented in Figure 1 and Table 1.

**Figure 2:**
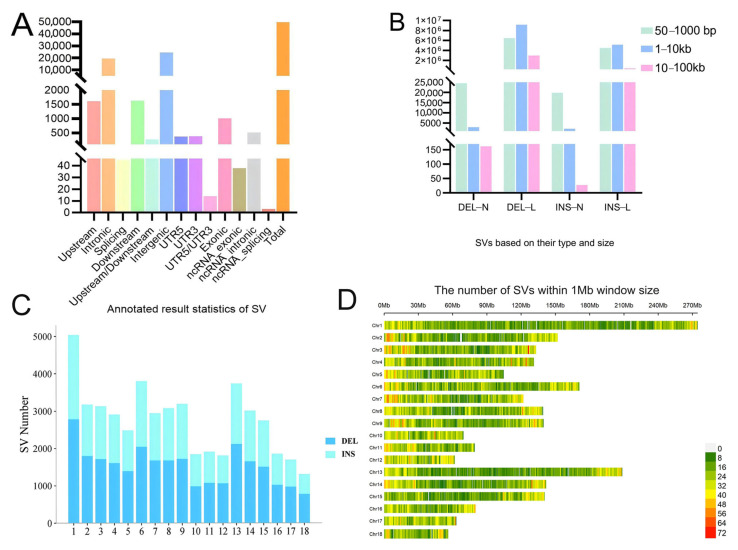
The structural variations (SVs) in Tibetan pigs. (**A**) Annotated result statistics of SV; (**B**) distribution of SVs based on their type and size. N: number; L: length. (**C**) Distribution of SVs on the autosomes, (**D**) distribution of SV site density in Tibetan pigs. Note: Upstream: the mutation is located in the 1 Kb region upstream of the encoding gene; Intronic: the mutation is located in the intron region of the coding gene; variation is located in the splicing sites of coding genes (including the 2 bp splicing interface); Downstream: the mutation is located in the 1 kb region downstream of the coding gene; Upstream/Downstream: the variation is located in the 1 kb region between adjacent coding genes. Intergenic: located in an intergenic region; UTR5: the mutation is located in the non-coding region of the 5′ end of the encoding gene; UTR3: the mutation is located in the non-coding region of the 3′ end of the encoding gene. UTR5/UTR3: the mutation is located in both the 5’UTR and 3’UTR regions of the encoding gene. Exonic: the mutation is located in the exon region of the gene; ncRNA_exonic: variation sites are located in the exon region of non-coding RNA; ncRNA_intronic: the variation occurs in the intronic region of a non-coding RNA molecule; ncRNA_splicing: the variation may be located in the splicing regulatory region of non-coding RNA; Total: indicates the total number of mutation sites.

Using SURVIVOR to merge SVs jointly detected by SVIM-asm and SYRI, a total of 30,807 deletions (DEL), 25,623 insertions (INS), 0 inversions (INV), and 0 duplications (DUP) were identified (Figure 2). After excluding SVs smaller than 50 bp, the distribution of the remaining SVs across each chromosome was analyzed. This filtered set, which comprised 22,008 high-confidence insertions and 27,639 deletions (hereafter collectively referred to as “SVs”), was used for all subsequent population-level analyses, including SV genotyping, the GWAS, and comparisons of SV distribution patterns (Figure 2C). Chromosome 1 exhibited the highest SV number, while chromosome 18 showed the lowest SV number, as illustrated in Figure 2. To better understand the distribution of SVs relative to chromosome size, we normalized the SV count by chromosome length and present the results as SV density (number of SVs per megabase, SVs/Mb) in Figure 2D. After this normalization, chromosome X exhibited the highest SV density, while chromosome Y showed the lowest. Notably, even though chromosome 1 had the highest absolute SV count, its density was not the highest, indicating that SV accumulation is not merely a function of chromosome length but may also relate to factors such as gene density, recombination rate, or chromatin structure.

**Figure 3:**
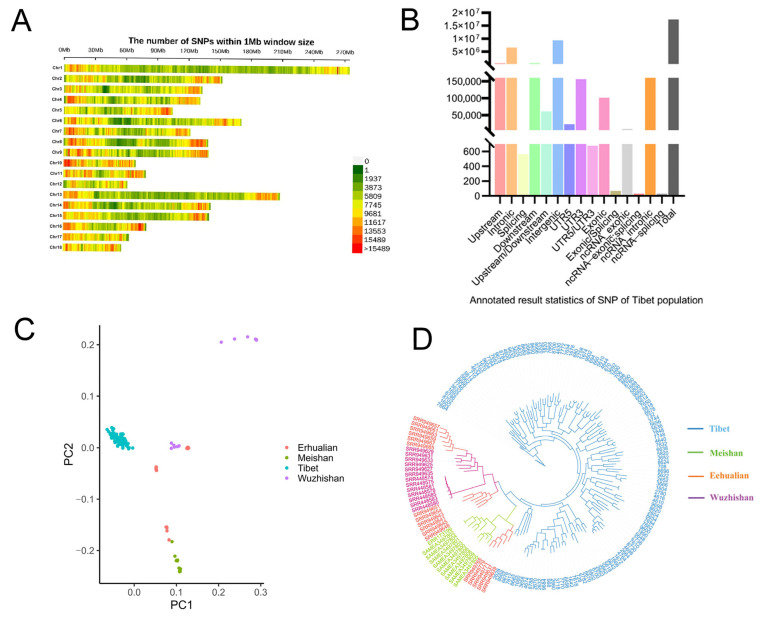
Genetic variation and population structure analysis in Tibetan pigs. (**A**) Distribution of SNP site density in Tibetan pigs; (**B**) annotated result statistics of SNPs of Tibet population; (**C**) results of principal component analysis for the four groups; (**D**) phylogenetic tree of four pig populations.

After quality control filtering, a total of 17,425,725 SNP loci and 165 individuals were retained for subsequent analysis. Tibetan pig SNPs were annotated using ANNOVAR [31] (Figure 3B). The distribution of SNPs across chromosomes is visualized in Figure 3A. The SNP distribution density was notably higher at chromosomal termini and comparatively lower in central regions.

The PCA results were visualized using the first two principal components (PC1 and PC2). As illustrated in Figure 3C, the Tibetan pig and Meishan pig populations each formed distinct, tightly clustered groups. In contrast, the Erhualian pigs exhibited population stratification, dividing into three subgroups: one subgroup clustered near the Meishan pigs, while the other two showed proximity to the Wuzhishan pigs. Similarly, the Wuzhishan pigs displayed stratification, with one subgroup adjacent to the Erhualian pigs and another subgroup distinctly separated from all other breeds.

The phylogenetic tree analysis revealed distinct clustering patterns: Tibetan pigs and Meishan pigs formed concentrated, independent groups, while Erhualian pigs segregated into three subgroups. One Erhualian subgroup clustered within the same branch as Meishan pigs, while the other two subgroups grouped with Wuzhishan pigs—flanking the Wuzhishan cluster on both sides. These findings align with the PCA results across all four populations (Figure 3D). However, the stratification observed in the Wuzhishan pigs within the PCA plot did not manifest in the phylogenetic tree topology.

### 3.2. Analysis of Selection Signals for Tibetan Pigs

**Figure 4:**
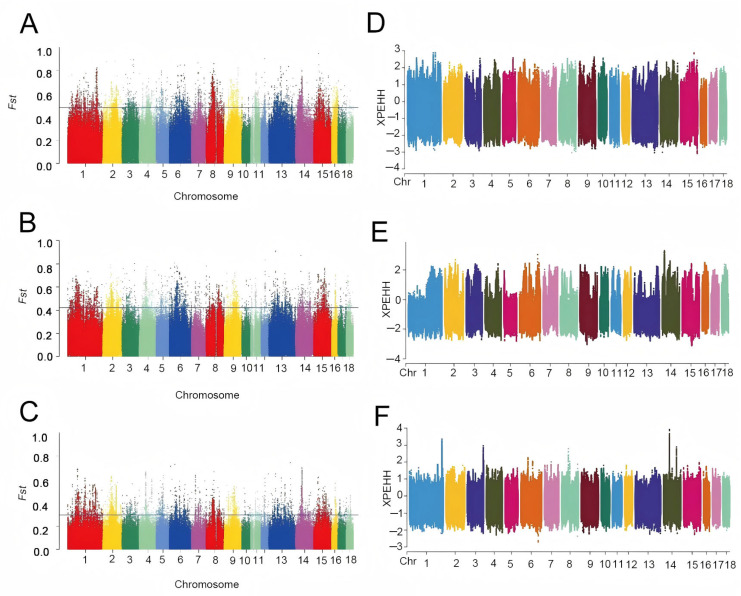
Selection signature analysis between Tibetan pigs and three domestic pig breeds. (**A**) FST analysis results of Tibetan pigs and Meishan pigs; (**B**) FST analysis results of Tibetan pigs and Erhualian pigs; (**C**) FST analysis results of Tibetan pigs and Wuzhishan pigs. (**D**) XPEHH analysis results between Tibetan pigs and Meishan pigs; (**E**) XPEHH analysis results between Tibetan pigs and Wuzhishan pigs; (**F**) XPEHH analysis results between Tibetan pigs and Erhualian pigs.

Comparative FST analysis between Tibetan pigs and Meishan pigs analyzed 432,458 genomic windows, with the top 1% windows annotated to identify 311 candidate genes. For Tibetan pigs versus Erhualian pigs, 432,459 windows were analyzed, yielding 320 annotated genes from the top 1% signals. Similarly, analysis of Tibetan pigs against Wuzhishan pigs covered 432,460 windows, with the top 1% windows annotated for 323 genes (Figure 4A–C).

In the XPEHH analysis comparing Tibetan pigs with Meishan, Wuzhishan, and Erhualian pigs, 1,847,766, 1,703,475, and 1,957,229 positively selected loci were detected in Tibetan pigs, respectively. Annotation of the top 1% loci identified 176, 154, and 151 candidate genes. To refine candidate gene identification, intersections of genes annotated by both methods (FST and XPEHH) were extracted: 12 overlapping genes for Tibetan vs. Meishan pigs, 9 for Tibetan vs. Wuzhishan pigs, and 12 for Tibetan vs. Erhualian pigs. Functional annotation revealed genes associated with cardiac function maintenance (*XIRP2*), lipid metabolism (*CACNA1A*), cartilage development (*COL11A1*), insulin release (*GIPR*), lipogenesis (*KSR2*), and vasodilation (*ADORA2A*). These genes may underlie Tibetan pigs’ adaptation to high-altitude environments (Figure 4D–F).

### 3.3. Distribution of ROHs of the Breeds

**Figure 5:**
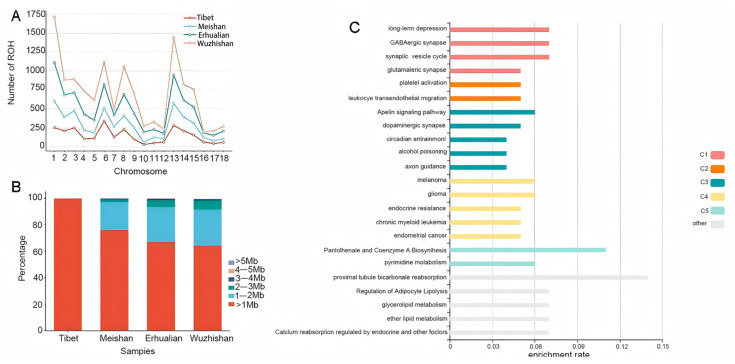
Genomic analysis of runs of homozygosity in four pig breeds. (**A**) Distribution of ROHs on the autosomes; (**B**) proportion of ROHs of different lengths in four pig breeds; (**C**) ROH annotated gene KEGG pathway enrichment.

ROH detection was performed on 124 Tibetan pigs, 13 Wuzhishan pigs, 17 Erhualian pigs, and 11 Meishan pigs. Among 165 individuals, 157 had ROHs detected (7 undetected: 2 Tibetan, 3 Wuzhishan, 2 Meishan). A total of 12,667 ROH segments were detected across the four groups, with Wuzhishan pigs having the most (3874) and Meishan pigs the fewest (2520). Across the 18 autosomes, the total and minimum numbers of ROH segments per chromosome were: Tibetan (124/22), Wuzhishan (838/97), Erhualian (423/27), and Meishan (515/94). Tibetan pigs had the most ROH segments on chromosome 6, while the other groups had the most on chromosome 1. Tibetan and Meishan pigs had the fewest ROH segments on chromosome 10, while Wuzhishan and Erhualian pigs had the fewest on chromosome 16; their distribution is shown in Figure 5A. Segments were categorized by length (<1 Mb, 1–2 Mb, 2–3 Mb, 3–4 Mb, 4–5 Mb, >5 Mb) (Figure 5B). The <1 Mb category was the most abundant in all groups and present on every chromosome, while the >5 Mb category was the least abundant. Subsequent analysis focused on the 124 Tibetan pigs, where <1 Mb segments were predominant (94.94%) and no segments > 3 Mb were detected. This may relate to their extensive rearing (semi-grazing limiting timely estrus detection, increasing natural mating) and strong activity in high-altitude grasslands, where enclosures are less restrictive, resulting in less selective breeding. The top 1% most frequent SNPs within Tibetan pig ROH segments were annotated, identifying 410 genes. KEGG enrichment analysis of these genes showed significant enrichment in pathways related to synaptic transmission, disease, and lipid metabolism (Figure 5C).

### 3.4. Statistical Results of Phenotypic Expressions of Reproductive Traits

**Figure 6:**
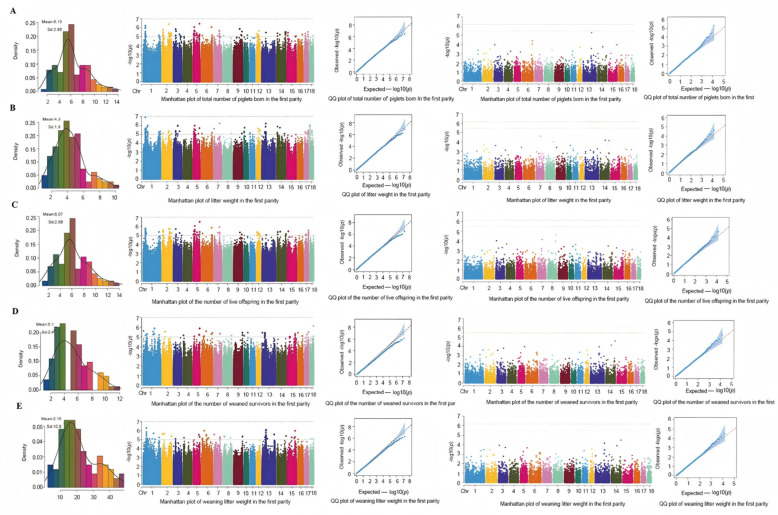
Concordant GWAS signals for litter traits from SNP and SV analyses in Tibetan pigs. (**A**) Total Number Born; (**B**) Weight of Total Number Born; (**C**) Number Born Alive; (**D**) Number of weaning survival; (**E**) Weaning Litter Weight. Note: (**A**–**E**) represent the phenotypic data statistics of each litter trait and the corresponding SNP and SV GWAS results from left to right.

As shown in Figure 6, Tibetan pigs exhibited considerable inter-individual variation across five reproductive traits (TNB, NBA, NPW, WTNB, WLW), with notable differences between maximum and minimum values. The mean values for litter-related traits were significantly lower than those of commercial pig breeds. Proximity between median and mean values indicated a balanced distribution of high and low litter sizes within the Tibetan pig population. Non-zero skewness (absolute values < 0.25) and kurtosis (>0 but near zero) across all traits revealed mild left/right distributional shifts, leptokurtic peaks (sharper than normal distribution), and slightly heavier tails, though overall distributions approximated normality.

Following genome-wide association analysis, the genome-wide significance threshold was calculated as 5.734 × 10^−10^ and the chromosome-wide threshold as 2.869 × 10^−9^, though no variants reached these significance levels. Given the absence of significant signals—a scenario paralleling a GWAS on peach fruit size, where suggestive signals (*p* < 1 × 10^−5^) were adopted [32]—we implemented a suggestive threshold of *p* < 1 × 10^−5^. For the Number Born Alive (NBA) trait, 102 suggestive loci were identified and annotated to 22 genes; for Weight of Total Number Born (WTNB), 106 loci were annotated to 24 genes; for Number of Piglets Weaned (NPW), 48 loci were annotated to 8 genes; for Weaning Litter Weight (WLW), 70 loci were annotated to 14 genes; and for Total Number Born (TNB), 121 loci were annotated to 23 genes. In SV-based association analysis, no significant SV was detected; thus, the top 20 most significant SVs per trait were annotated as candidate regions, ultimately identifying six candidate genes (Figure 6): *SCGB1A1*, *HAO2*, *NRG4*, *MTUS2*, *FGF12* and *C8A.*

## 4. Discussion

### 4.1. The Chromosome-Level Genome Assembly and Structural Variant Map for Tibetan Pigs

The genome, encompassing all genetic information within an organism, directs diverse biological functions and records the evolutionary history of species. Rapid and accurate determination of the base sequence in an organism’s genomic DNA is crucial for life sciences research. We performed de novo assembly of the Tibetan pig genome using stLFR sequencing data, with TGS-GapCloser and RagTag employed for gap filling. Post-scaffolding, the genome exhibited significant improvements in metrics including N50, N90, L50, L90, and largest contig, resulting in a more contiguous assembly. Published genomes of Meishan pigs and Chenghua pigs are 2.5 Gb and 2.6 Gb in size, with largest contigs of 107.29 Mb and 209.54 Mb, and completeness of 96.8% and 96.2%, respectively [33]. Our assembled Tibetan pig genome is smaller than these, likely due to their use of third-generation sequencing technologies, which offer superior resolution in complex genomic regions. While stLFR assembly supplemented with ONT data and reference-guided scaffolding significantly enhanced the largest contig metric, limitations in overall completeness persist. Nevertheless, BUSCO completeness assessment was comparable, indicating robust assembly quality. Structural variant (SV) detection via genome alignment is widely applied. Early genomes based on short-read sequencing had limited SV detection capacity. Using SVIM-asm and SYRI, we identified 22,008 insertions and 27,639 deletions between the Tibetan pig genome and the Duroc reference. To ensure SV genotyping accuracy, three Tibetan pigs were randomly selected to evaluate depth effects. Truvari analysis showed an average accuracy of 0.9735 at 10× depth, approaching 100% at 20× depth (maximum sequencing depth). Previous genetic investigations into Tibetan pig adaptation have been almost exclusively focused on single-nucleotide polymorphisms (SNPs) and small indels [6,10]. Our study presents the first systematic catalog and analysis of genome-wide SVs in Tibetan pigs, thereby expanding the scope of investigable genetic variations underlying their unique phenotypes.

### 4.2. Population Genomics and Selection Signals for High-Altitude Adaptation

To decipher the genetic architecture of high-altitude adaptation, we conducted comparative population genomics using whole-genome resequencing data from 124 Tibetan pigs and three geographically and phenotypically distinct lowland Chinese breeds (Meishan, Erhualian, Wuzhishan). Selection signal analysis identified key divergent genes between Tibetan and Meishan pigs: *XIRP2* (cardiac functional compensation) [31]; *GIPR* [34] and *CACNA1A* (lipid metabolism) [35]; and *COL11A1* (tendon fibril assembly influencing mobility) [36]. Compared with Erhualian pigs, critical candidate genes included *KSR2* (energy metabolism via AMPK pathway) [37] and *ADORA2A* (vasodilation/angiogenesis enhancing oxygen delivery) [38]. These genes synergistically support enhanced cardiac function, efficient energy utilization, and locomotor adaptation in hypoxic environments. Analysis of runs of homozygosity (ROHs) revealed high-frequency selection signals enriched in lipid metabolism pathways (e.g., lipolysis regulation, glycerolipid metabolism). Core candidate genes *LIPE* (lipase regulating glycerol release) [39], *PNPLA2* (triglyceride hydrolysis; [40]), *DGAT1* (triglyceride synthesis affecting fat deposition) [28] and *MGLL* (monoacylglycerol breakdown for energy mobilization) [41] form a lipolytic homeostasis regulatory network. Their high conservation [27] and dual roles in fat storage/mobilization likely optimize energy storage efficiency and thermal insulation in Tibetan pigs under unstable food supply and extreme cold. Novelty relative to prior studies: Earlier selection studies in Tibetan pigs often used single lowland breeds. Our targeted comparison with breeds of extreme fecundity (Meishan) and distinct climatic adaptation (Wuzhishan) allowed us to disentangle signals specific to plateau life from general porcine genetic variation. This approach revealed novel candidate genes like *KSR2*, implicating whole-body energy homeostasis as a crucial component of adaptation beyond immediate oxygen sensing.

### 4.3. Decoding the Genetic Architecture of Reproductive Traits via Integrated GWASs

Reproductive performance represents a critical limiting factor in Tibetan pig production. To investigate its genetic basis, we conducted genome-wide association studies (GWASs) for five litter traits using both SNP and structural variant (SV) markers genotyped in 124 phenotyped sows. Our integrated analysis revealed a network of candidate genes associated with distinct reproductive stages. The GWAS based on SNPs and SVs identified 338 loci associated with reproductive traits. Gene annotation and pathway enrichment analysis indicated that for Total Number Born (TNB), *ATP23* and *IFT27* are key candidate genes: *ATP23* influences sow estrus and ovulation by regulating energy metabolism and cellular stress responses, while *IFT27* participates in reproductive regulation by maintaining sperm morphology and function, with its deficiency linked to male infertility. For Number Born Alive (NBA), associated genes encompass multidimensional functions in embryonic development, nutrient metabolism, and immune regulation: *ADGRB3* affects embryonic brain development via neuronal migration [42]; *TMPRSS6* regulates iron absorption to support hematopoiesis [32]; *ADGRA1* maintains metabolic homeostasis through PI3K/AKT and MEK/ERK pathways [43]; *IL2RB* and *C8A* enhance maternal immune protection via T-cell proliferation regulation and complement system activation, respectively [44]; and *FGF12* plays critical roles in central nervous and cardiac development [45]. Weight of Total Number Born (WTNB)-associated genes (*PRKAG2*, *PTPRN2*, *GSX1*, and *HAO2*) synergistically regulate energy metabolism and growth: *PRKAG2* (an AMPK subunit) drives energy allocation; *PTPRN2* promotes nutrient accumulation by stabilizing insulin secretion; *GSX1* accelerates development via growth hormone-releasing hormone activation [46]; and *HAO2* optimizes energy utilization through fatty acid oxidation [47]. For Number of Piglets Weaned (NPW), core genes *TRDN* and *SCGB1A1* enhance piglet adaptation to high-altitude hypoxia by stabilizing cardiac rhythm and strengthening pulmonary immune defense, respectively. For Weaning Litter Weight (WLW), *SLIT2* supports tissue development via angiogenesis promotion [48]. As a key member of the TGF-β superfamily, *BMP6* is highly expressed in ovarian granulosa cells, oocytes, and the corpus luteum. It participates in the regulation of reproductive processes by modulating follicular development, ovulation, and progesterone synthesis [10]. Previous studies have confirmed its significant role in regulating litter size traits in pigs. Given the high-altitude hypoxic environment in which Tibetan pigs survive, it is hypothesized that hypoxia may downregulate *BMP6* expression, thereby affecting follicular development and luteal function. The differential expression of this gene in Tibetan pigs may represent an adaptive mechanism to cope with hypoxic stress while maintaining baseline reproductive capacity. *BMP6* drives skeletal growth [49], while *NRG4* improves metabolic efficiency by inhibiting hepatic lipogenesis [50]. Additionally, studies have suggested that *NRG4* may exert a protective role under hypoxic conditions by activating the PI3K/Akt signaling pathway, a crucial cellular survival pathway [51]. *MTUS2* influences overall growth potential by regulating cardiac and muscle development [52]. Novelty relative to prior studies: This study represents the first GWAS for reproductive traits in Tibetan pigs that explicitly incorporates structural variations (SVs) alongside SNPs. This complex regulatory network—spanning optimization of energy metabolism, assurance of embryonic development, enhancement of immune defense, and specialization of organ functions—collectively underpins Tibetan pigs’ reproductive adaptability in high-altitude hypoxic environments and provides critical molecular targets for genetic improvement of plateau livestock.

## 5. Conclusions

In conclusion, this study systematically deciphered the molecular mechanisms underlying high-altitude adaptation and reproductive traits in Tibetan pigs by integrating multi-omics and multidimensional genetic analyses. We successfully constructed a chromosome-level genome assembly (2.25 Gb, N50: 136.5 Mb) with significantly optimized coverage (94.16%) and BUSCO completeness (95.7%), providing a precise reference framework for plateau animal genomics. Regarding hypoxia adaptation mechanisms, we discovered that *XIRP2* enhances cardiovascular function through myocardial stress response reinforcement, while *CACNA1A* regulates lipid homeostasis; concurrently, *LIPE* and *DGAT*1 optimize energy reserves via lipometabolic equilibrium, collectively forming a tripartite genetic strategy of cardiovascular reinforcement, metabolic remodeling, and energy utilization coordination. For unique reproductive performance, we revealed a molecular regulatory network comprising 13 core genes (*IL2RB*, *PRKAG2*, *BMP6*, etc.) that ensures plateau reproductive efficiency through a tripartite “embryonic development–maternal metabolism–immune defense” mechanism. ‘*ADGRB3*/*FGF12*’ promotes embryonic neural development; ‘*PRKAG2*/*NRG4*’ regulates energy allocation via the AMPK pathway; and ‘*IL2RB*/*C8A*’ enhances maternal–fetal interface immune protection. The Tibetan pig genome assembly and key genes identified herein not only provide novel perspectives for deciphering high-altitude adaptive evolution but also offer crucial theoretical support for genetic resource conservation, marker-assisted breeding, and improved breeding of plateau-specific varieties. Future studies could further validate candidate gene regulatory mechanisms through functional experiments to facilitate translational application of superior genetic resources in Tibetan pigs.

## Figures and Tables

**Table 1:** Statistics of the Tibetan pig genome assembly results.

Stat Type	stLFR Denovo	Rag Tag
	Contig Length (MB)	Contig Length (MB)
N50	1.51 MB	133.31 MB
N90	0.19 MB	60.24 MB
L50	425	8
L90	2038	17
GC (%)	41.74	41.74
Largest contig	15.27 MB	271.77 MB

Note: N50: The length of the last contig added when the sum of lengths reaches 50% of the total contig length. N90: The length of the last contig added when the sum of lengths reaches 90% of the total contig length. L50: The minimum number of contigs required when the sum of lengths reaches half the length of the genome. L90: The minimum number of contigs required when the sum of lengths reaches 90% of the genome length.

## Data Availability

All relevant data are contained within the article. The original contributions presented in the study are included in the article; further inquiries can be directed to the corresponding authors.

## Supplementary Materials

The following supporting information can be downloaded at: https://www.mdpi.com/article/10.3390/ani16030509/s1, Table S1: Use of group information.
