# Supplementary material for: Genomic Signatures Underlying Environmental Adaptation and Reproductive Traits in the Tibetan Pig
Source: Animals (Basel). 2026 Feb 5;16(3):509. doi: 10.3390/ani16030509 (PMC12896720; doi:10.3390/ani16030509)
Supplement: Supplementary file 1 [file animals-16-00509-s001.zip › animals-4007108-supplementary.pdf]

Schedule

Table S1 Use of Group Information

Data number	Individual number	Population	colony
SRR949637	EHL1	EHL	Erhualian pig
SRR949639	EHL2	EHL	Erhualian pig
SRR949641	EHL3	EHL	Erhualian pig
SRR949643	EHL4	EHL	Erhualian pig
SRR949645	EHL5	EHL	Erhualian pig
SRR949648	EHL6	EHL	Erhualian pig
SRR949651	EHL7	EHL	Erhualian pig
SRR949654	EHL8	EHL	Erhualian pig
SRR949657	EHL9	EHL	Erhualian pig
SRR949659	EHL10	EHL	Erhualian pig
SRR949661	EHL11	EHL	Erhualian pig
SRR949663	EHL12	EHL	Erhualian pig
SRR949665	EHL13	EHL	Erhualian pig
SRR949667	EHL14	EHL	Erhualian pig
SAMEA3497800	MS1	MS	Meishan pig
SAMEA3497801	MS2	MS	Meishan pig
SAMEA3497802	MS3	MS	Meishan pig
SAMEA3497803	MS4	MS	Meishan pig
SAMEA3497804	MS5	MS	Meishan pig
SAMEA3497805	MS6	MS	Meishan pig
SAMEA3497806	MS7	MS	Meishan pig
SAMEA3497808	MS8	MS	Meishan pig
SAMEA3497809	MS9	MS	Meishan pig
SRR448575	WZS1	WZS	Wuzhishan pig
SRR949625	WZS2	WZS	Wuzhishan pig
SRR949627	WZS3	WZS	Wuzhishan pig
SRR949629	WZS4	WZS	Wuzhishan pig
SRR949631	WZS5	WZS	Wuzhishan pig
SRR949633	WZS6	WZS	Wuzhishan pig
SRR949635	WZS7	WZS	Wuzhishan pig

Note: The Tibetan pig data is from sequencing, and other populations are derived from public databases.
